# Palliative care delivery in residential aged care: bereaved family member experiences of the Supportive Hospice Aged Residential Exchange (SHARE) intervention

**DOI:** 10.1186/s12904-020-00633-x

**Published:** 2020-08-17

**Authors:** Rosemary Frey, Sophia Barham, Deborah Balmer, Michal Boyd, Jackie Robinson, Merryn Gott

**Affiliations:** 1 School of Nursing, Faculty of Medical and Health Sciences University of Auckland, 85 Park Road, Grafton, Auckland, New Zealand; 2 Division of Health Sciences, University of Otago, Dunedin, New Zealand

**Keywords:** Palliative care, Bereaved families, Residential aged care, Older adults

## Abstract

**Background:**

The supportive hospice aged residential exchange (SHARE) is a new model of palliative care education that has been designed for residential aged care. The goal of SHARE is to help clinical staff improve palliative care within residential aged care facilities and to improve specialist palliative care nurses’ knowledge and skill to care for frail older people.

**Method:**

The experiences of 18 bereaved families concerning the palliative care journey (both at the start and finish of a one-year implementation of SHARE) were explored using semi-structured interviews.

**Results:**

Three themes were important to bereaved families’ experience: communication with staff, systems of care, and hospice involvement. Sub-themes indicating changes in these three components of care between the start and finish of SHARE was identified. A fourth theme highlighted challenges (relationship with GP, staff shortages, and turnover) that continued across SHARE.

**Conclusion:**

Findings indicated that SHARE benefited families (improved communication and support) through the end of life journey of their relatives, but challenges remained.

## Background

New Zealand, like many developed countries, has an ageing population. By 2035, it is expected that almost 25% of New Zealand’s population will be over the age of 65 [1]. Increasing age often includes multi-morbidity and frailty and an increasing need for more comprehensive and complex care, especially at the end of life [2]. From 2003 to 2007, 38% of deaths in New Zealanders aged over 65 were in residential aged care (RAC) facilities [3]. In New Zealand (Aotearoa), the term RAC covers a range of long-term aged care services, based on level and type of care need including 24-h hospital care, rest home care, and dementia/psychogeriatric care [4]. The RAC ownership model within New Zealand is dominated by large privately owned facilities [3]. At the end of 2019, New Zealand had 39,000 residential aged care beds [1]. This number is only expected to increase, with an estimated 52,000 RAC beds being required by the end of the decade [1]. Overall, if current patterns continue, the majority of deaths for people over the age of 85 will be in RAC, accompanied by increased complex geriatric, frailty, and multi-morbidity care requirements being more complex as time progresses [5, 6]. As a result, there is a growing burden for New Zealand RAC facilities to provide good quality palliative and end of life care [7].

Palliative care is defined as care for a person with a life-limiting illness that aims to optimise the quality of life for the person, as well as support their whānau (family) caregivers both during illness and after death [2]. Palliative care is one component of an overall health care plan and may be more needed than a purely treatment focused plan at the end-of-life [8]. Palliative care is delivered by both generalists as a part of standard clinical practice by any healthcare professional and by specialist palliative care professionals who have undergone specialist training in palliative care [2]. Hospices provide both in- and out-patient, palliative care for those with a terminal or life-limiting diagnosis [9]. However, for many older adults in RAC, palliative care will be required in the context of multi-morbidity, in particular chronic non-malignant illnesses [10]. It could be argued the RAC facilities are one of the main providers of palliative care, often without the support of Hospice [3, 10, 11]. For RAC facilities to deliver high-quality palliative care, facilities require adequate resources, adequately trained staff, and access to specialist palliative care [2]. However, RAC facilities in New Zealand are faced with the challenges of organisational conditions characterised by increasing workload, low staffing levels, and high staff turnover [12].

In New Zealand, as in other developed countries, the growing patient population means there is an urgent need to invest in the development of “generalist palliative care” [13]. As defined by the New Zealand Ministry of Health [13] generalist palliative care is care “provided for those affected by life-limiting illness as an integral part of standard clinical practice by any health care professional who is not part of a specialist palliative care team”. However, health professionals often feel ill-prepared to provide effective palliative care, especially surrounding the discussion of death and dying, and being able to communicate effectively with families during the end of life period [4, 14, 15]. Advance Care Planning (ACP) is one example of these hard to have conversations, which involves shared planning between the RAC resident, their family and the RAC health professionals on the important values and wishes for the end of life, and includes incorporating these into the resident’s care plan [4]. Having healthcare professionals unskilled in these areas can compromise the care provided to residents and their families, as well as create added stress during the end of life [14, 15].

Traditional educational interventions (e.g. short training courses, online modules) have had varied success [10, 16, 17]. In New Zealand, healthcare professionals within RAC settings work within a context of too few staff and high turnover [17]. The burnout that results from these conditions, can negatively impact the uptake of these didactic courses [18]. Furthermore, didactic courses have proven to be unsuccessful in producing sustained knowledge transfer [4, 18, 19]. The Supportive Hospice and Aged Residential Exchange (SHARE) intervention sought to fill the gap in palliative care education for RAC staff.

SHARE involves the following activities led by a hospice nurse specialist from the hospice: 1) weekly visits over 1 year to each RAC. At the initial visit, all residents were reviewed to identify those with palliative care needs using the Clinical Frailty Scale [20] and the Supportive and Palliative Care Indicators Tool (SPICT) [21] in collaboration with a RAC nurse. The list of residents with palliative care need became the focus of discussion for subsequent visits; 2) clinical coaching and role modelling of palliative care and gerontology skills and knowledge (this is a reciprocal process of shared learning between hospice and RAC staff); 3) in partnership with staff in RAC learning needs concerning palliative and end of life care were identified and a program of teaching developed by hospice; 4) guidance and role modelling of advance care planning conversations with RAC nurses and 5) debriefing with RAC staff surrounding resident deaths [22].

SHARE builds on the strengths of current palliative care practice in RAC by combining new learning with existing skills and knowledge. SHARE also improves specialist palliative care nurses’ knowledge and skill in gerontology by working collaboratively with the RAC nurses. A qualitative analysis of written reflections by the specialist palliative care nurses indicated that sustained relationships with RAC staff (registered nurses, healthcare assistants) was a key factor supporting the implementation of this palliative care educational intervention [4]. The SHARE model of care thus provides a mechanism to support knowledge exchange between hospice staff and clinical care staff to improve palliative care delivery within RAC facilities.

The SHARE intervention was implemented over a year in 20 urban RAC facilities across two district health boards (DHBs) in one urban centre. This paper forms part of a larger mixed-method evaluation of SHARE which included quantitative assessments of staff palliative care delivery confidence, a records review of residents identified as benefitting from a palliative approach to care, as well as qualitative interviews with staff, general practitioners (GP’s), facility managers and bereaved families [23].

### Role of families

Families play a key role in a resident’s ongoing care and also act as the closest link to residents’ views of care received [24, 25]. Previous research has identified shortfalls in the management of the transition to palliative care in RAC from the family perspective [26]. In particular, research has identified RAC staff members having difficulty communicating information about residents’ likely prognosis to family members [27], as well as engaging families in care planning [26]. Additionally, families have expressed dissatisfaction due to a general practitioner’s inaccessibility and/or changes in the general practitioner responsible for the care [28]. These difficulties create barriers to the formation of a ‘partnership’ between staff and family which could enhance the quality of care [29], of particular importance at the end of life. The SHARE intervention was designed to improve palliative care delivery for residents and their families. Given the key role that family members have in a resident’s care, this study explored changes in bereaved family perceptions of palliative care delivery. This study sought to identify potential areas to be addressed during subsequent implementations of SHARE.

## Method

### Aim

The study aimed to describe bereaved family member’s experiences of palliative care for their relative in RAC facilities implementing SHARE. This study makes up a part of a larger mixed-methods quasi-experimental evaluation of the SHARE intervention in 20 RAC facilities. The evaluation utilised both quantitative (survey, records review) and qualitative methods (interviews and focus groups) to assess the impact and sustainability of the intervention.

### Design

This study views participants as sources “situated knowledges” [30, 31] reflecting patterns of meanings and beliefs not preordained by an existing theory [32]. A qualitative descriptive design was adopted to explore the experiences of 18 bereaved family members regarding palliative care delivery for their relative in RAC facilities implementing SHARE. Bereaved family participants provided a situated account of the facilitators and contextual challenges to palliative care delivery observed during SHARE implementation [32]. Comparison lies at the heart of qualitative analysis [33]. Drawing on the constant comparative method outlined by Boeije [34] interviews from two different groups were compared concerning the experience of a specific phenomenon (SHARE). The important question posed is: What do bereaved families before SHARE say about certain themes and what do families after SHARE have to say about the same themes?

### Process

Bereaved family members were recruited through the 20 RAC facilities that implemented SHARE across two urban district health boards (DHB’s). Out of the 20 facilities contacted, nine facilities responded with contact details for bereaved family members interested in participating. All participants had a family member die in the past year in RAC. Bereaved families were recruited from RAC facilities at two different time points in SHARE implementation: RAC less than 1 month into the implementation of SHARE (start) and RAC facilities who had implemented SHARE for 1 year (finish). The total number of interviews conducted was shaped in part through “developing the range of relevant conceptual categories, saturating (filling, supporting, and providing repeated evidence for) those categories,” and fully explaining the data [35].

Participants were interviewed using a semi-structured interview schedule developed during the pilot study [22]. The interview schedule explored psychosocial impacts including satisfaction with life, communication skills, grief, loss, and survivor guilt/shame (see Supplementary File). Interviews of approximately 60 min of duration were audio-recorded with participant permission. Interviews were transcribed verbatim by a transcriptionist who had signed a confidentiality agreement. All data collection took place between November 2017 and April 2019. All bereaved family participants were assured of anonymity, confidentiality, and their right to withdraw from the study at any time. Ethics approval for the study was obtained from the university ethics committee Ref # 020075.

### Analysis

Transcripts were uploaded to the analysis software QSR NVivo 12. SB conducted a reflexive thematic analysis, developing themes, and sub-themes related to the characteristics associated with family perceptions of palliative care delivery [36]. First, immersion in the data was achieved by reading transcripts several times and creating memos based on general themes constructed. Then, open coding was utilised and several codes were generated across the data set. Codes were labelled using terms used by the participants or relevant to the literature. Codes were then collapsed into broader categories. The list of broader categories was then condensed further into main categories and then-candidate themes. Candidate themes were reviewed against the transcripts and refined to reflect patterns of shared meaning represented in the transcripts. The constant comparative method was then used to identify whether SHARE duration shaped the experience of the previously identified themes. In other words, “What did bereaved families at the start of SHARE say about developed themes and what did bereaved families at the finish of SHARE have to say about the same themes?” through the concrete experiences of the bereaved families [37]. In line with previous research [38] coding of all transcripts was completed to develop themes before comparisons between the two groups of interviews were undertaken. Analyst triangulation [39] of the results was established through discussion with project co-authors who hold a variety of expertise (gerontology, palliative care, social psychology, ethnography).

## Results

### Participants

The qualitative methodology used in this study was to gain an in-depth understanding of bereaved family experiences of care for their relative in the RAC setting. In keeping with that approach, demographic data were collected in the context of the interviews. A brief overview is provided. Eight interviews with bereaved family members (7 women, 1 man) were conducted before SHARE. Interviewees were most often between the ages of 70–79 and the majority [8] identified as NZ European and reported Christianity as their religion [4]. Five of the decedent residents had diagnoses of dementia (Table 1). Ten post-intervention bereaved family member interviews were completed. The majority of participants were of European ethnicity [8] and female [7]. Half of the participants were between the ages of 70–79 years (Table 1). Half of the deceased relatives post-SHARE had a diagnosis of dementia. The majority of deceased residents both pre and post SHARE had been ill for less than 1 year (Table 1). To protect the confidentiality of both participants and the residential aged care facilities, quotes from participants were assigned pseudonyms based on a colour (e.g. emerald, garnet, etc.).

**Table 1:** Demographic Overview of Interview Participants

	Start-SHARE (*n* = 8)		Finish-SHARE (*n* = 10)	
	Frequency	%	Frequency	%
**Age group**				
40–49	1	12.5	1	10.0
50–59	2	25.0	2	20.0
60–69	1	12.5	2	20.0
70–79	3	37.5	5	50.0
80+	1	12.5	0	0
**Gender**				
Female	7	87.5	7	70.0
Male	1	12.5	3	30.0
**Ethnicity**				
NZ European	8	100	8	80.0
Māori	0	0	1	10.0
Asian	0	0	1	10.0
**Language**				
English	8	100	8	80.0
Te Reo Māori	0	0	1	10.0
Chinese(Mandarin or Cantonese)	0	0	1	10.0
**Religion**				
No Religion	3	37.5	4	40.0
Christian	4	50.0	4	40.0
Buddhist	0	0	1	10.0
Judaism	1	12.5	0	0
Spiritualist	0	0	1	10.0
**Diagnosis Dementia**				
Yes	5	62.5	5	50.0
No	3	37.5	5	50.0
**Length of Illness**				
< 7 days	1	12.5	3	30.0
1–4 weeks	5	62.5	2	20.0
> 1 month, < 1 year	2	25.0	4	40.0
1 year or more	0	0	1	10.0

### Themes

**Fig. 1:**
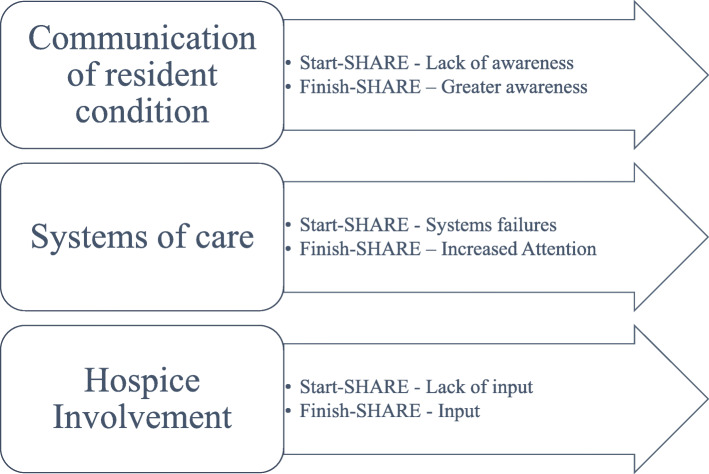
Bereaved Families Experiences of Palliative Care Delivery in SHARE Residential Aged Care Facilities: Themes and Sub-themes

Three themes outlined key components of palliative care that were important to relatives’ experience: communication with staff, systems of care, and hospice involvement. Comparisons in the above themes between the experiences of bereaved families at the start of SHARE and the finish are represented as subthemes (Fig. 1). A fourth theme, entitled ‘challenges’ outlined issues that persisted across SHARE. Subthemes included relationship with GP’s, staff shortages, and turnover. These issues were perceived by families as continuing barriers to achieving better palliative care for their relatives.

### Communication of resident condition

#### Start-SHARE

##### Lack of awareness

A key part of the SHARE intervention was teaching nurses how to effectively communicate with families about the transition to a palliative care approach and what to expect. Some family members described not finding out their family member was approaching the end of life until days before their relative’s death.

*Right, did the staff explain to you what might happen, the process of his dying? Did they talk to you about that, you might expect this or he might do this?*

*No, nothing like that.*

***Interviewer: So you had no idea what was going to happen, or how?***

*No, none at all, none at all.* (Emerald)

#### Finish-SHARE

##### Greater awareness

At the end of SHARE, however, while most of the relatives had no knowledge of any pathways (e.g. Liverpool Care Pathway) that their relatives might be on, they did know when they were dying. Many family members recalled making advance care plans, alongside an registered nurse [RN] and their relative. Many also recall being told their relative was heading towards the end of life. For many families, this allowed them to organise family before their relatives died and created an environment that was less distressing before death. The following quote outlines the planning and communication between RAC staff and the family member that allowed for a better-perceived death.

*But you know, they took me aside and made sure that I understood what the, what the process was and how it would work. And to make sure that I actually understood - I guess that’s before them starting the morphine, it was pretty obvious by then… And they got her stable and I wasn’t going to go home, but they got her stable and she was quite good. And I went home, I’m one of five so I contacted all my siblings and said, look Mum’s not in a good space but she’s stable, but I just think you need to know this. You know, if you’re available to come in tomorrow that would probably be a good idea to see her.* (Alba)

### Systems of Care

#### Start-SHARE

##### Systems failures

Systems of care sometimes let families down, especially when it came to caring for people with dementia:

*He had a fall in the bedroom, that’s right, he fell and hit his mouth on the dressing table. And of course, there was blood and everything, so I took him down to [urgent care], that was it, and they said they couldn’t do the plastic surgery. So we went out to [hospital], I took him out to [hospital], so they examined him out there. They said they couldn’t do anything that night because he was on Warfarin, so we were sent home from [hospital] and then told to come back the next morning at seven. Am I allowed to say haha?* (Emerald)

#### Finish-SHARE

##### Increased attention

Many families felt like their relative was well cared for at the end of SHARE. All commented that the actual end of life moments were peaceful and calm. This is a change from the start of SHARE and may be attributable to the increased attention that has been placed on the end of life processes and improved communication. Staff were well versed in the care of their relative, as well as relaying meetings with doctors and changes in medication: One family commented on the increased attention and open lines of communication:

*They were good. Very good. Like change of medicine or anything, I’ll always get a phone call...*

*Oh, the Doctors gone to see [resident] and we’re changing his medication. Or we’re doing something. So, no they, they kept me up in touch all the time.* (Azure)

One daughter commented on the quality of care and the peace of mind gained:

*I mean I really do feel that she had the best care and the best dying experience…. And it makes me feel so good to be able to go to bed at night and she’s, she’s had that*. (Violet)

### Hospice involvement

#### Start-SHARE

##### Lack of input

Another key aspect of SHARE was a collaboration between Hospice Nurse Specialists and RAC staff, particularly RNs and formal caregivers. At the start of SHARE, family members did not report Hospice having any input in their relative’s care, or that this was even an option for them.

***Interviewer: And hospice wasn’t involved with your Dad?***

*No.*

***Interviewer: No, no, okay.***

*No, we didn’t have any hospice people at all.*

***Interviewer: And you weren’t asked about it?***

*No, no we weren’t. I don’t know, is that an option, can you get that? I wouldn’t have a clue. No, we were never, that was never offered or asked.* (Grey)

#### Finish-SHARE

##### Input

Having the SHARE intervention integrated into palliative care provision within RAC facilities meant there was a collaboration between Hospice Nurse Specialists and RAC staff. As a result, some families were aware of Hospice involvement in their care, although this was still primarily for residents with cancer diagnoses. More generally, however, the presence of the Hospice Nurse Specialists seemed to aid families in feeling more aware of the process of dying and offered them extra support for what would happen after death. In this way, families felt looked after and involved in their relative’s passing.

*According to Mrs. H, she remember [Nurse X] and some other Nurses, they are coming from Hospice, have talked to her about [resident] is dying, what she need to do. They have people talk to her about making an arrangement for the funeral. She even remembers they have explained to her a few days before [resident] died they are going to give him a small dose of… to five milligrams of morphine, how they going to care for him. So those parts she was very much involved and have a conversation.* (Navy)

### Continuing challenges to the culture of care

#### Relationship with GP

Overall the relationship with the GP was a concern. One participant was so angry with the GP’s attitude towards her and her father that she made an official complaint. This seemed to be the result of the doctor and nursing staff not communicating well and this resulted in the GP writing an apology to the daughter. Another felt that the GP just went through the motions and did not show any concern for her mother’s welfare:

***Interviewer: On a scale of 1-5?***

*She would be on the lower [end], she might have been an efficient person, but mum couldn't stand her. She was absolutely useless in end of life care; she didn't care less about mum.*

*Oh we complained, my daughter complained badly about the doctor’s attitude, etc, etc, so we’ve got a big, long letter of apology from her, and why she did what she did.* (Indigo)

The above respondent talked about the lack of communication between the staff and the GP. There appeared to be no syringe driver on site and there were difficulties getting one as well as the medication to put in it; finally, this was completed through advice from the hospice GP and the facility.

#### GP communication

GP’s communication skills continued to be variable in quality from the families’ perspective. One participant had moved her mother from one facility (A) to another facility (B). In facility (A) the resident had been on iron tablets for a long time. The GP in facility (A) had not communicated clearly with the daughter concerning the treatment decisions, leaving her concerned. In Facility (B) the GP felt further investigation was required. The quote illustrates the damaging result of an inadequate discussion of the care options with the mother and daughter.

*As soon as she got into facility (B) the Doctor there said her bloods aren’t right, which I’d known from the one up in facility A, but they just kept giving her iron tablets. And she and the Doctor at facility (B) said, no, we need to find out why her bloods aren’t right. So they sent her to Hospital, so within a week of her coming down here she was sent to the Hospital.* (Garnet)

Many did not see the GP at all and relied on communication and information through the nurse manager. Some participants found their lack of interaction with the GP worrying and disturbing:

***Interviewer: Did you see the Doctor at all?***

*Nope. Had no faith in him.*

***Interviewer: Did you not?***

*Yeah. There were, there were many times I asked, when does he visit this place? Can I make an appointment please to see him? I’d like to sit with him and my sister. The only, the only medical professions I had, had face to face consultation with was the hospital. [Family member] and I said, yay, we’d like to speak to her Doctor. Nothing.* (Cyan)

#### Fewer staff

A private funding model for the facilities in this study created increasing challenges for the implementation of SHARE. Three of the participants talked about staffing levels, and two had relatives at the same facility where there was an obvious shift in the numbers of staff and also the culture of care. Both interviewees noted that staff suddenly had less time for their relatives, and while they did their job satisfactorily, it was completed quickly with less time allocated and less conversation. One relative specifically went in to feed his father because he knew that the staff did not have time:

*Even his, like at the end he needed to be fed. So I’d go and make, I always went over and well I left here about 10:30, 11 so I was there for lunch, give the staff some time. And they, they appreciated it. You know.*

***Interviewer: Did you notice that? That they were pressed for time.***

*Oh yeah, well I know that, so that’s why I did it. I know that they have, well I know, 60 was it, 65 there and there’s three girls at lunch trying to get them all fed. So they haven’t got time to feed each and everyone that required it.* (Azure)

*Definitely fewer staff. And that was a large part of it too. They always, you know, would be like, oh we’re so understaffed and, I didn’t often have a need to ring the bell but on a couple of times that I did, you know, it might be a toileting issue that I, I needed help with… And in situations like that, you would wait you know, to the point where like just sit there Mum and don’t move. And I’d go off looking for somebody. They hadn’t come to answer the bell.* (Alba)

#### Turnover

Some commented that the staff would change continually, especially for those residents who had been at the facility over several years. This became confusing for next of kin, who dealt with it by by-passing the health care assistants and going to the nurse manager for any information:

*Because they were so different, different people there all the time and I’d ask where’s such and such? Where’s so and so? And, and resident would be doing the same, and it got to the stage where we didn’t want to ask, you know. So if we had a problem we’d go and see the manager.* (Cyan)

## Discussion

Findings indicated that good communication can play an integral role in the family experience of palliative care for their relative [40]. Poor communication has been linked to detrimental outcomes for families including increased difficulty in decision-making and a lack of preparedness for a relative’s death, impacting on bereavement [41, 42]. At the start of SHARE and consistent with previous research [43], bereaved family members of RAC residents, felt uninformed about the residents’ health and felt that they were not given information on what to expect at the end of life. In line with previous research [26, 44], bereaved family perceptions of quality of care appeared improved at the finish of SHARE by earlier communication among residents, family members, and health professionals about prognosis, options for care, and assessment of support and coping resources.

Interviews after a year of SHARE indicated that more communication and collaboration between Hospice and RAC facilities led to positive responses by families regarding the quality of care for their relatives [4, 8]. Reflecting previous research [8] a poor relationship between hospice and RAC facilities was a source of family concern before SHARE. After a year of SHARE, the collaboration between hospice and RAC promoted bereaved family members’ confidence in the care of their relatives. Post-SHARE none of the families expressed dissatisfaction with the communication processes and all commented that the actual end of life moments were peaceful and calm. This is a change from interviews before SHARE which indicated a need for improved staff communication. This change may be attributable to the increased attention that has been placed on end of life and improved documentation by the nursing staff, as well as modelling of advance care planning conversations by the hospice nurse specialists. Recall that after SHARE many family members reported making advance care plans, alongside an RN. However, while SHARE includes debriefing of staff following a death, future implementation of the intervention should include extending RAC support to relatives post-bereavement as well as RAC wide post-death rituals and practices [45].

Communication with GPs, as perceived by bereaved family continued to be an area of difficulty. A recent study looking at the experiences of family members as their relatives transitioned to palliative care supports these results [24], that better communication at the end of life, especially with GPs, would have improved their relative’s experience at the end of life. However, evidence suggests that for families or RAC residents, contact with GP’s is limited [10]. There are many potential reasons for bereaved families’ expressed dissatisfaction in communication with GPs. Firstly, time and cost restraints preclude communicating at length with families [46, 47]. Many of the cases of poor communication and care from GPs from families in this study focused on how communication was handled, and the extent to which GPs showed respect for residents and families. Recent research has found that training in the care of dying RAC residents consists primarily of informal mentoring among GP’s [47]. Literature suggests the lack of palliative care training available for GPs, compounded with time pressures, leads to a lack of effective communication skills at the end of life, and can negatively impact the level of care felt by residents and their families [48]. It should be noted however, that SHARE was an intervention directed at improving the palliative care knowledge and skills of the RAC nursing and health care assistant staff and not GP’s. Future implementations should incorporate greater involvement of GP’s from the start of SHARE.

While care quality continued to be seen to be good, bereaved families continued to express concerns about low staffing levels and staffing changes after a year of SHARE. Poor staffing levels, high turnover, and associated time pressures can threaten the continuity of care within RAC [49]. Since relationships between residents, families, and staff are at the heart of good palliative care, time pressures and increasing staff turnover created barriers to the formation of enduring relationships between families, residents, and staff. Continuing family concerns over the stability of the workforce may impact upon both confidence in RAC staff and the sustainability of the intervention. The continued presence of a hospice nurse specialist mentor takes on even greater importance given the growing resourcing and staffing issues within the sector. Relationships between hospice and facility staff, and consequently facility staff and residents and their families are seen as key to maintaining the successes of the intervention.

### Strengths and limitations

Only the views and experiences of bereaved family members are included in these findings. The views of registered nurses, GPs, healthcare assistants, and hospice nurse specialists have been presented elsewhere [4, 47, 50]. Nevertheless, the experiences of these bereaved family members provide both a unique insight into the benefits and challenges to the implementation of SHARE [51]. Interviews were carried out within 12 months of a relative’s passing to both lessen the burden on the interview participant, and to ensure they could recall details from their relative’s passing [52]. A further limitation was the absence of Māori and Pacific Island representation. It is well established that Māori and Pacific peoples suffer historic and institutional health disparities [53]. In a New Zealand context, Māori and Pacific Island perspectives and experiences are imperative to ensuring the SHARE project has equitable outcomes.

## Conclusion

Good palliative care delivery requires both resident and family-centred care [8]. Findings from this study support the view that bereaved families perceived both improved communication and increased support through the end of life journey of their relatives after SHARE. Issues in communication with GP’s and bereaved family perceptions of the negative impacts of staffing shortages on relative’s care continue to present challenges to SHARE implementation. Both are complex issues that must be addressed to improve palliative care for RAC residents and families alike.

## Supplementary information

**Additional file 1.**

## Data Availability

De-identified datasets used and/or analysed during the current study are available from the corresponding author on reasonable request.

## Abbreviations

ACP: Advance care planning
DHB: District health board
GP: General practitioner
RAC: Residential aged care
SHARE: Supportive hospice aged residential exchange
SPICT: Supportive palliative indicators tool
