# Supplementary material for: Palliative care delivery in residential aged care: bereaved family member experiences of the Supportive Hospice Aged Residential Exchange (SHARE) intervention
Source: BMC Palliat Care. 2020 Aug 17;19:127. doi: 10.1186/s12904-020-00633-x (PMC7433142; doi:10.1186/s12904-020-00633-x)
Supplement: Supplementary file 1 — Additional file 1. [file 12904_2020_633_MOESM1_ESM.docx]

**Supportive Hospice and Aged Residential Exchange**

**Part One**

**Interview Guide for Bereaved Families**

Interview guide for families/whanau

• Introductions

• Answer any questions participants have

• Explain the purpose of the interviews

• Take consent

• Explain the recording device

**General**

1. What was her/his main illness (such as heart failure, cancer, diabetes, etc.)

2. How long had she/she been ill before she died?(Tick one only)

- She was not ill — she died suddenly
- Less than 24 hours
- One day or more, but less than one week
- One week or more, but less than one month
- One month or more, but less than six months
- Six months or more
- One year or more

1. Can you tell me the experience you had with the facility surrounding your relative/whanaunga’s death and the care they received at that time?

- How much of the time was she treated with respect and dignity by the staff at the last residential care facility she stayed in? (Tick one only)
- Always
- Most of the time
- Some of the time
- Never
- Don’t know

Please explain

1. How did the staff show (or not show) they were treating her with respect and dignity?

Explain

1. During the last three months of her/his life, while she/he was in the residential care facility, how well was her/his pain relieved? (Tick one only)

- Does not apply – she/he did not have any pain
- Completely, all of the time
- Completely, some of the time
- Partially
- Not at all
- Don’t know

Please explain

1. What does palliative care mean to you? Did the staff talk to you about palliative care?
2. Did the facility staff use a specific end of life procedure that you knew about, for example. Liverpool Care Pathway or Last Days of Life Pathway?
3. Can you explain what you think would be a “good dying journey”? Was there anything that was not helpful and stopped a good dying journey?
4. Was hospice involved in the dying journey in any way?
   1. Were you involved in the decision to involve hospice? How was the decision made to involve hospice

**Communication**

1. Please rate your level of satisfaction on a scale where (1 = "Not at All Satisfied"; 2 = "Not Very Satisfied"; 3 = "Somewhat Satisfied"; 4= "Very Satisfied"; 5 = "Completely Satisfied") on the following questions:

How satisfied are you that the doctor(s) explained things relating to your relative's illness in a straightforward, honest manner *during the past month*?

Explain

How satisfied are you that the doctor(s) explained things relating to your relative's illness in a way you could understand *during the past month*?

Explain

How satisfied are you that you received consistent information about your relative's condition from all the doctors and nurses looking after him or her *during the past month*?

How satisfied are you that you received updates about your relative's condition, treatments, test results, etc. in a timely manner *during the past month*?

Explain

How satisfied are you that the doctor(s) listened to what you had to say *during the past month*?

How satisfied are you with discussions *during the past month* with the doctor(s) about where your relative would be cared for (in hospital, at home, or elsewhere) if he or she were to get worse?

Explain

**Support**

1. Did you and your whanau feel support from the facility throughout the dying process? Was the support right for you and your whanau? What would you have changed?
2. Were you and your whanau offered additional support (ie chaplain, social worker, kaumatua, counsellor) by the facility or anyone else (?) while your relative/ whanaunga was dying? Would you have benefitted from support?
3. If you were to give advice, tautoko, to other family members experiencing a dying process with a family member what would you say
4. Is there anything else you would like to talk about that has not been covered so far? Anything that was left unresolved or left you feeling unhappy?

APPROVED BY THE UNIVERSITY OF AUCKLAND HUMAN PARTICIPANTS ETHICS COMMITTEE ON 26 October 2017 for 3 years, Reference Number 020075
